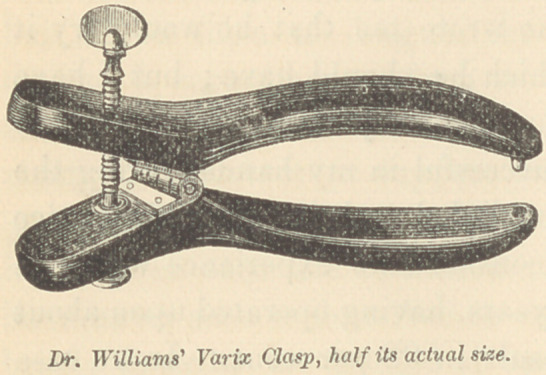# An Improved Varix Clasp for the Radical Cure of Varicocele

**Published:** 1879-05

**Authors:** T. W. Williams

**Affiliations:** Milwaukee, Wis.


### ARTICLE III.

### AN IMPROVED VARIX CLASP FOR THE RADICAL CURE OF VARICOCELE. By T. W. WILLIAMS, M.D., Milwaukee, Wis.

*Of the Disease.*—Varicocle, as is well known, depends upon an enlargement, or varicose condition of the veins which form a large part of the spermatic cord. It is sometimes situated on the right side, but generally on the left, owing, it has been stated, to the absence of a valve where the left vein enters the emulgent, which subjects it to greater pressure from the superincumbent column of blood. Whatever may be the cause, the fact is that the walls of these veins do give way, become relaxed, as it were, and distended, bulging out in places into little pouches, giving the vessel, when full of blood a knotty appearance. The whole vein, in old cases, is dilated, enlarged, tortuous, cordy and knotty. The disease is one very prevalent, and although it has been known to exist for years without any serious results, my observation has convinced me that these are exceptional cases, and that in the majority of instances the disease results in impotency and wasting of the testicle, and sooner or later impairs the general health by constant nervous irritation, inducing dyspepsia, dragging sensations in the groins, lumbago, or pain in the back, and a general depression of the system, despondency, gloomy forebodings, and hypochondria.

*Of the Treatment.*—We cannot, by any means known to medicine, restore these veins to their normal healthy condition; but we can do the next best thing, which is to occlude them, that is, cut off the circulation in them. The palliative treatment, which consists in cold bathing, counter irritants, and wearing a suspensory bandage, seldom affords more than temporary relief. A permanent and radical cure is effected only by the occlusion of the veins. The barbarous practice of removing part of the scrotum, as recommended by Sir Astley Cooper; including the veins and half of the scrotum in a ligature; exposing the veins and tying them, wearing a lever truss, which was worse than useless, etc., etc., were formerly resorted to. Ricord was the first to improve on this treatment, by substituting the sub-cutaneous ligature. Dr. Gross still further simplified and improved the operation by using only one ligature. This operation for varicocele, although considered perfectly safe, and undoubtedly effective of a radical cure, is not without objection. To be performed by a competent surgeon, its cost (no small item to numerous patients), is considerable, while the loss of time, occasioned by the patient being confined to his room two or three weeks, is both painful and tedious. The operation by the ligature is also excruciatingly painful. "Pain is a great evil," as Dr. Gross says, and if we can accomplish the same end without it, we thereby gain a material advantage.

Many cases of varicocele are caused by a relaxed, pendulous condition of the scrotum, which does not support the testicles, but allows them to drag on the cord. In early stages of the disease, I sometimes perform an operation for retrenching the scrotum, by means of an *elastic ligature* which shortens it sufficiently to cause it to support and sustain the testicle. I have performed this operation several times with the best results. I have never seen the operation performed by any one else, and have never seen it described or recommended. I will, therefore, briefly describe my method of operating, which is very simple. I gather up sufficient of the relaxed scrotal tissue on the affected side to shorten the sac, so as to cause it to lift the testicle and support it sufficiently to prevent any tension of, or dragging on the cord. Around this superfluous portion I slip an elastic ligature sufficiently strong to compress it and arrest the circulation in the vessels of the part. I then pass a small needle, threaded with fine silver wire, through the occluded portion just *below* the ligature, and bring it through the scrotum again just *above* it, forming a tight loop around the ligature, secured by twisting the ends of the wire together. I then leave it to cut through and slough off, which it does with very little swelling.

The operation is not attended by any considerable pain, and if it is performed in the early stages all further trouble is avoided. Even after the occlusion of the cord by the ligature or clasp, many patients are obliged to wear a suspensory, and the further operation of shortening of the scrotum is advisable; as also in relaxed conditions of the sac, even where no disease exists.

*Operation with the Varix Clasp.*—But the operation which I consider preferable to all others for the radical cure of varicocele, is that of occlusion of the diseased veins by means of an improved varix clasp of my own. I do not claim originality for this operation, and am aware that a similar operation was recommended many years ago, but by whom I have not the data at hand at this moment to say. I think an instrument for this purpose is figured in an old edition of Pancoast's Surgery, published from sixteen to twenty years ago. I do not think, however, the operation ever received much favor, and have never seen it referred to in any medical literature on the subject which has come under my own observation; and when I sent one of my instruments to Dr. S. D. Gross, some fifteen years ago, it seemed to strike him as a good idea, and he wrote me that he would try it upon the first favorable case which he should have; but I have never heard anything from him on the subject since.

The operation has been so successful in my hands during the last fifteen years, that I have concluded to bring it to the notice of the profession. I have had considerable experience with the affection during the last fifteen years, having operated upon about 200 cases with a successful result in 98 per cent. In no case has any fatality attended the operation, and in the three or four cases in which it has failed, I attribute the failure to the slipping of the cord. As the clasp was originally constructed, as shown in the engraving, the pin at the end of the blade was simply a blunt knob. Its object was to prevent the cord from slipping out of the blades upon the application of pressure by the thumb-screw. In the improved instrument, as now made by Messrs. Tiemann & Co., of 67 Chatham St., N. Y., this pin is made longer and sharper, so as to pass *entirely through* the scrotum, thus effectually preventing this accident.

The clasp is made of hard rubber, beveled from the center of the blades outward; the blades are 5 Cm. long, from the point to the hinge, and are approximated by a thumb-screw. I consider the clasp a great improvement upon the old method of operating. It acts on the principle of the ligature, without the pain and inconvenience attending that operation. The result is the same as if a skillful ligation had been performed, and is obtained with much less pain and loss of time. It possesses other advantages which the ligature does not, viz.: the slight inflammation and ulceration of the external skin which it produces, causes the scrotum, which is always relaxed, to contract and become shortened; while it is perfectly free from the danger of inflammation and peritonitis, which sometimes follow the use of the ligature. Owing to the *rapidity of the cure*, and *freedom from internal suppuration*, the dangers and inconvenience of the ligature are avoided. The function of the testicle remains unimpaired, which is not always the case after other operations.

*Mode of Applying the Clasp.*—The application of the instrument is so simple and easy that the physician will have no difficulty in accomplishing it. The spermatic artery and vas deferens should not be included in the clasp, as our dealings are entirely with the veins. The artery and vas deferens can be easily distinguished by the touch when taken between the thumb and forefinger. They are united, and form one cord, which is about the size of a quill, and has a hard cordy feel, totally different from the soft vascular feel of the veins, and the pulsation of the artery can be distinguished as a rule. After separating the cord from the veins with the thumb and forefinger of the left hand, by pushing it over towards the other testicle, let the patient lie down so as to empty the veins (still keeping them separated), then apply the clasp so as to include and compress the veins about an inch

*Dr. Williams' Varix Clasp, half its actual size.*

above the testicle, turning the thumb-screw until the veins are completely strangulated. Should the blades of the clasp when closed leave too much room between them to compress the veins sufficiently, as may happen when the veins are thin, one or more thicknesses of chamois skin or rubber may be put between the blade and the skin. This is rarely necessary, however, as the blades of the clasp approach within one-sixteen of an inch of each other. The pin at the end of one blade passes *completely* through the scrotum, between the vas deferens and artery on one side and the enlarged veins on the other. This is necessary to prevent the veins from slipping out of the clasp when the pressure is applied. The pain of this operation is much slighter than one would suppose, and lasts only a few minutes, after which the parts compressed become deadened.

*The After Treatment.*—By leaving the clasp on five days the veins become clogged up with coagulated lymph, and are thus permanently closed, so that when the instrument is removed, the circulation is not re-established. The instrument usually causes more or less dull aching pain for a few hours, and the pressure on the external skin causes it to become sore, but it heals up in a short time after the removal of the clasp. The lower part of the vein, at the bottom of the scrotum, becomes more or less swollen and distended; the blood, however, is in time re-absorbed, and, if necessary, the swelling may be poulticed with ground flaxseed, or bread and milk, to hasten its departure, after the removal of the clasp. Four or five days is sufficient to effect a cure. A bandage of some kind should be worn to prevent the clothes rubbing the clasp off; and after its removal a suspensory bandage should be worn until all swelling and tenderness subside. The diet should be light, and the recumbent posture maintained as much as possible while wearing the clasp. The patient may, however, move about the room, and go down and up stairs to his meals or to the closet. In a month after removing the clasp, it can be seen whether the cure is perfect or not; in some cases, from various causes, it may be necessary to make a second application of the instrument; but this will be very rare, and then will be owing to the first application not including all the veins. A radical cure may be relied on in all cases.

The cut represents the varix clasp as originally made. The only alteration I have made in it, was to make it a little heavier and stronger, and have the pin so constructed as to pass entirely through the scrotum. They may be obtained from Messrs. Tiemann & Co., made after my own model. They are known as "Williams' Varix Clasps," but are not a *patented* or proprietary article.

---

### ARTICLE IV.

## Figures and Tables

**Figure f1:**